# Interactions that know no boundaries

**DOI:** 10.1107/S2052252518002713

**Published:** 2018-02-26

**Authors:** Michael E. Wall

**Affiliations:** a Computer, Computational and Statistical Sciences Division, Los Alamos National Laboratory, Los Alamos, NM 87545, USA

**Keywords:** diffuse scattering, intermolecular correlations, LLM models

## Abstract

Diffuse scattering provides evidence that variations are correlated across molecular boundaries in macromolecular crystals.

Deviations from an ideal crystal lead to diffuse scattering (DS) intensity, both between and beneath the Bragg peaks in diffraction patterns (Guinier, 1963 ▸). First characterized using simple ionic crystals in early studies of X-ray diffraction (Lonsdale, 1942 ▸), DS has a rich history (Welberry & Weber, 2016 ▸) and is a well established technique in small-molecule crystallography (Welberry, 2004 ▸). DS studies in macromolecular crystallography began more recently (Phillips *et al.*, 1980 ▸) and now the potential for obtaining information about protein motions is fueling the growing interest in DS (Meisburger *et al.*, 2017 ▸).

As noted in a previous **IUCrJ** commentary (Keen, 2016 ▸), accurate modeling of small-molecule DS requires not only information about the variations of individual molecules or unit cells, but also information about the correlated variations in a more extended environment. Similarly, macromolecular DS studies indicate the importance of modeling interactions across unit-cell boundaries in normal-modes models (Riccardi *et al.*, 2010 ▸), as well as the molecular dynamics models (Wall, 2018 ▸) of macromolecular diffuse scattering that are shown in this issue. The liquid-like motions (LLM) model (Caspar *et al.*, 1988 ▸), in which the correlated variations are modeled as if the crystal were a soft homogeneous material, explains the overall DS pattern in several protein crystals (Caspar *et al.*, 1988 ▸; Clarage *et al.*, 1992 ▸; Van Benschoten *et al.*, 2016 ▸; Wall, Clarage & Phillips, 1997 ▸; Wall, Ealick & Gruner, 1997 ▸). However, the consequences of including intermolecular interactions for the accuracy of the LLM model were not clear until now.

In this issue, Peck and co-workers (Peck *et al.*, 2018 ▸) investigate the importance of intermolecular interactions by assessing the accuracy of two alternative versions of an LLM model (Caspar *et al.*, 1988 ▸) (Fig. 1 ▸). In the original version of the model, the correlations extend across molecular boundaries (Fig. 1 ▸ *a*). In this case, the diffuse intensity is derived from the squared crystal transform, which is sharply peaked. In a modified version of the model, correlations terminate at the molecular boundary (Fig. 1 ▸ *b*). In this case, the diffuse intensity is derived from the squared molecular transform of the asymmetric unit (in the cases considered, a single molecule), which is continuous in reciprocal space. In both cases, the transform is blurred; shorter correlation lengths correspond to a larger scale blurring of the transform. Both models are optimized to maximize the agreement with the data, enabling a well controlled comparison.

To be consistent with the state of the art (Meisburger *et al.*, 2017 ▸), three-dimensional diffuse datasets were used for the comparison, obtained from crystalline cyclophilin A (CypA) (PDB entry 4yuo; Fraser, 2015 ▸), WrpA (PDB entry 5f51; Herrou & Crosson, 2015 ▸) and alkaline phosphatase (PDB entry 5c66; Peck *et al.*, 2017 ▸). The CypA data were the subject of a prior DS study (Van Benschoten *et al.*, 2016 ▸) and the others were newly analyzed for this study, providing valuable additions to the currently limited amount of available macromolecular DS data. For all three datasets, the original LLM model, which includes intermolecular correlations, was substantially more accurate than the modified model in which molecules are independent. Instances of elastic network models (ENMs) (Bray *et al.*, 2011 ▸) and ensemble models (van den Bedem *et al.*, 2009 ▸) were tested, but since these models were only narrowly explored and did not incorporate prior insight into their use for diffuse scattering calculations (Levin *et al.*, 2007 ▸, Riccardi *et al.*, 2010 ▸), the tests were inconclusive. Models of independent rigid-body motions (Moore, 2009 ▸) also were compared; their accuracy was similar to that of the independent LLM model, providing additional evidence for the importance of intermolecular interactions in macromolecular DS.

As illustrated in this issue (Peck *et al.*, 2018 ▸; Wall, 2018 ▸), for a small number of cases, DS studies provide insight into what is really going on in macromolecular crystals, beyond what can be discerned using Bragg analysis. However, DS data collection and processing is less well developed in comparison with the Bragg analysis, and model accuracy is still lacking. Until general insights are available from a larger number of cases, it would be wise not to dismiss any individual approach to analyzing the data. As for the Bragg data, each DS data point is influenced by the whole system; therefore we can expect that the entire DS model will need to be sufficiently accurate before the atomic details of macromolecular movements can be revealed.

## Figures and Tables

**Figure 1:**
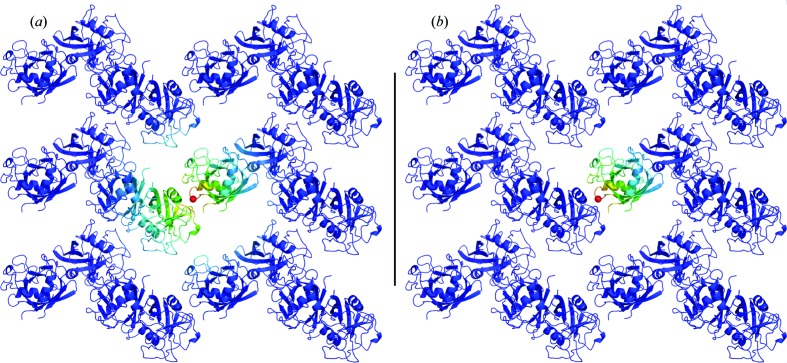
Alternative models of correlated displacements in a liquid-like motions model, compared by Peck *et al.* (2018 ▸). Each panel depicts a 2 × 3 unit-cell section extracted from the (010)–(001) layer of crystalline cyclophilin A [PDB entry 2cpl (Ke, 1992 ▸), used in Peck *et al.* (2018 ▸)]. The *b* axis is aligned with the vertical direction, and the *c* axis with the horizontal. Each *P*2_1_2_1_2_1_ unit cell contains four copies of the protein that arrange into a sawtooth in this projection. The strength of correlation with an atom near the center (Asp26 Cα, highlighted as a red sphere) is indicated using a heat map. The correlation decays exponentially with distance, according to a liquid-like motions model (Caspar *et al.*, 1988 ▸). (*a*) The original model, in which correlations extend to atoms on different proteins. (*b*) A modified model, in which correlations are limited to atoms on the same protein. Peck and coworkers (Peck *et al.*, 2018 ▸) found that diffuse scattering data for this and two other systems favor model (*a*) over model (*b*). The figure was created using *PyMol* (https://sourceforge.net/projects/pymol/).
